# Review of *in vivo* optical molecular imaging and sensing from x-ray excitation

**DOI:** 10.1117/1.JBO.26.1.010902

**Published:** 2021-01-01

**Authors:** Brian W. Pogue, Rongxiao Zhang, Xu Cao, Jeremy Mengyu Jia, Arthur Petusseau, Petr Bruza, Sergei A. Vinogradov

**Affiliations:** a Thayer School of Engineering at Dartmouth, Hanover, New Hampshire, United States; b Geisel School of Medicine at Dartmouth, Lebanon, New Hampshire, United States; c Stanford University School of Medicine, Department of Radiation Oncology, Palo Alto, California, United States; d University of Pennsylvania, Perelman School of Medicine, Department of Biochemistry and Biophysics, Philadelphia, Pennsylvania, United States; e University of Pennsylvania, School of Arts of Sciences, Department of Chemistry, Philadelphia, Pennsylvania, United States

**Keywords:** Cerenkov, emission, fluorescence, phosphorescence, radioluminescence, scintillation

## Abstract

**Significance:** Deep-tissue penetration by x-rays to induce optical responses of specific molecular reporters is a new way to sense and image features of tissue function *in vivo*. Advances in this field are emerging, as biocompatible probes are invented along with innovations in how to optimally utilize x-ray sources.

**Aim:** A comprehensive review is provided of the many tools and techniques developed for x-ray-induced optical molecular sensing, covering topics ranging from foundations of x-ray fluorescence imaging and x-ray tomography to the adaptation of these methods for sensing and imaging *in vivo*.

**Approach:** The ways in which x-rays can interact with molecules and lead to their optical luminescence are reviewed, including temporal methods based on gated acquisition and multipoint scanning for improved lateral or axial resolution.

**Results:** While some known probes can generate light upon x-ray scintillation, there has been an emergent recognition that excitation of molecular probes by x-ray-induced Cherenkov light is also possible. Emission of Cherenkov radiation requires a threshold energy of x-rays in the high kV or MV range, but has the advantage of being able to excite a broad range of optical molecular probes. In comparison, most scintillating agents are more readily activated by lower keV x-ray energies but are composed of crystalline inorganic constituents, although some organic biocompatible agents have been designed as well. Methods to create high-resolution structured x-ray-optical images are now available, based upon unique scanning approaches and/or *a priori* knowledge of the scanned x-ray beam geometry. Further improvements in spatial resolution can be achieved by careful system design and algorithm optimization. Current applications of these hybrid x-ray-optical approaches include imaging of tissue oxygenation and pH as well as of certain fluorescent proteins.

**Conclusions:** Discovery of x-ray-excited reporters combined with optimized x-ray scan sequences can improve imaging resolution and sensitivity.

## 1 Introduction

Optical molecular imaging and sensing from x-ray excitation utilizes a fundamentally different type of interaction and sensing approach to excite optical reporters in biological tissues and detect and localize the emission. The biological utility and goals of x-ray-based sensing to better understand tissue physiology and pathophysiology are the driving motivations that underpin technical advances in this area. The major benefits of x-rays as a probe excitation source are (i) the high penetrance and (ii) the wide availability and acceptance of x-ray sources in biomedical imaging. When compared to other methods of molecular sensing in tissue, the strengths of x-ray-based molecular sensing may be less clear, because this methodology only began emerging during the last decade.^1^^–^^3^ However, the ability to sense through tissue using traditional x-ray sources, while still using optical molecular contrast, presents potential advantages in depth penetrance and spatial resolution. Optical sensing provides superior molecular sensitivity to x-ray-based contrast methods, because x-ray contrast is generally based upon the photoelectric effect, with peak attenuation in the keV energy range,^4^ and x-ray contrast agents usually need to be present in high levels, near millimolar quantities in tissue making them only useful for imaging blood volume and leakage. The field of x-ray-based molecular sensing benefits from an extraordinarily large range of detectors and sensors for optical emission that have sensitivity to the single photon level, making the detection side of the sampling potentially very efficient.

**Fig. 1:**
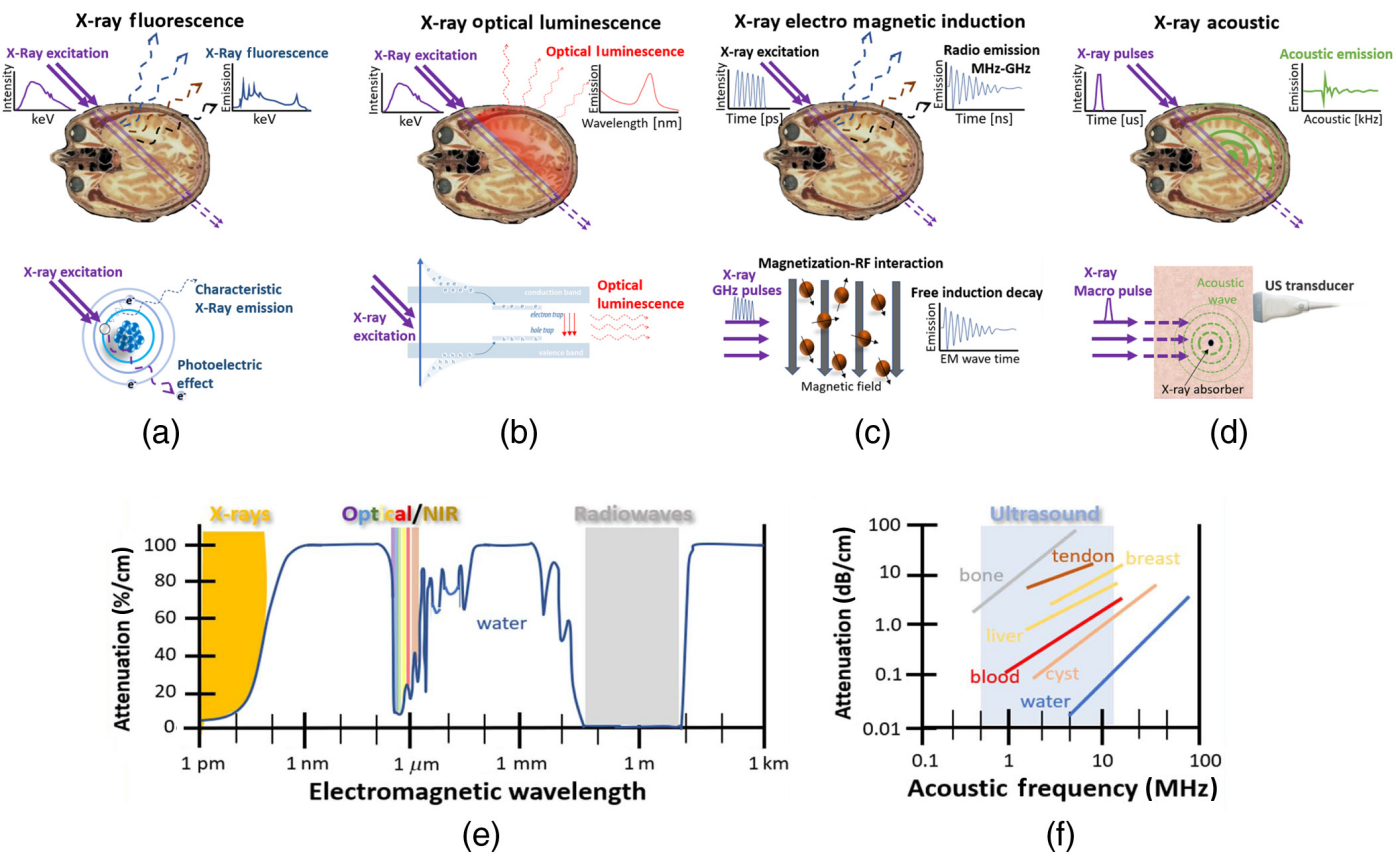
Schematic of the different detection schemes for probes, including (a) XRF where x-rays are both the excitation and emission, (b) x-ray optical luminescence where the output emission signal is optical from either scintillation or Cherenkov processes, (c) x-ray electromagnetic induction where there is either nuclear moment or electromagnetic change induced in the tissue, such that the output is a radiofrequency signal, and (d) x-ray acoustic where the output is induced by localized heating causing an ultrasound transient. As follows in (e) the attenuation spectrum of water is shown for electromagnetic radiation and in (f) the attenuation spectrum of acoustic frequencies is shown in tissue.

Perhaps the most scientifically enticing part of this methodology is the concept of utilizing one radiation source (i.e., x-rays) as the excitation probe, combined with another radiation type as a signal. This conceptual framework for developing a hybrid imaging modality is illustrated in Fig. 1 for several possible emission radiation types. X-ray activations with emission via pathways that are detectable through tissue are illustrated, including (a) x-ray-induced fluorescence, (b) x-ray-induced optical luminescence (the focus of this review), (c) x-ray-induced electromagnetic induction, and (d) x-ray-induced acoustics. While not all these approaches are reviewed extensively here, the ideal characteristics of such a hybrid method include:

i. unique molecular probe, able to bind with high affinity to biological targets;
ii. high contrast or specificity via high signal-to-background ratio;
iii. an emission signal type that can be detected with high signal-to-noise ratio; and
iv. an excitation radiation with high penetrance for imaging through tissue (1/μ≈d, where μ is the exponential attenuation coefficient and d is tissue thickness).

The emission signal types are illustrated in Fig. 1, with the attenuation values shown in Figs. 1(e) and 1(f).

In the case of optical emission, the single largest attraction is that optical molecular probes comprise arguably the most developed and diverse group of sensors for biological imaging. There are thousands of optical probes, and a number of them are commercially available for use in preclinical imaging. Consequently, preclinical optical imaging systems are the most widely used for whole body animal imaging,^5^ and imaging of tissue function and pathology with many types of optical stains is widely used both *in vivo* and *ex vivo*. The second major attraction for optical emission detection is its very high sensitivity, down to the single photon level. However, because of tissue scattering and absorption, there is exponential attenuation of optical signals with depth into tissue, although imaging through several centimeters of tissue is possible. The combination of a scanning x-ray excitation with detection of optical emission can circumvent the resolution limitations of diffuse optical imaging, while having the advantage of optical sensitivity and broad selection of optical imaging agents. Alternatively, combinations of optical sensing with other structural imaging tools, such as ultrasound, MRI, or computed tomography (CT), are also a commercially available paradigm.

**Fig. 2:**
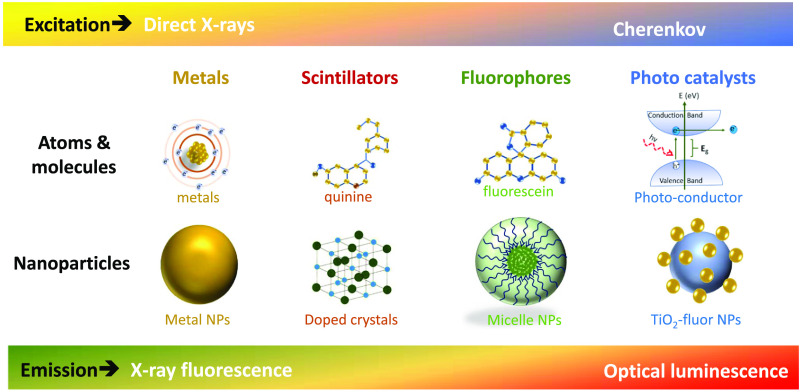
Illustration of the four major categories of probes possible with x-ray excitation, roughly categorized in terms of their capabilities for (upper bar) direct x-ray excitation or by secondary Cherenkov emission and (lower bar) by their emission capabilities transitioning from XRF to x-ray luminescence. Each category has both molecular (top row) and nanoparticle (bottom row) forms which are x-ray active.

The choices of x-ray sources, type, and mode of detectors are also quite diverse. The energy range of the x-rays needed depends upon the type of probe and its physical interaction mechanisms, as outlined in Fig. 2. Interaction mechanisms range from low keV energy x-rays, where the interaction with the probe is via the photoelectric effect, up to low MeV energies, where generation of Cherenkov radiation is the dominant mode to excite the probe. Overall, the following choices need to be considered: (i) x-ray source, (ii) molecular probe, (iii) emission detector, and (iv) the methodology of image recovery. These topics are reviewed as follows, with the focus on the advantages and disadvantages of existing approaches and on how the aforementioned choices are interconnected. As the technology progresses, it is imperative to think about the motivations, driving factors, and capabilities. This review begins with a historical summary from the origins of x-ray fluorescence (XRF) methods towards x-ray optical luminescence methods, with a focus on molecular sensitivity and utility, and then ends on a focus of what is needed for advancing the field capabilities.

## 2 X-Ray Fluorescence to X-Ray Optical Luminescence

The basic principles of using x-rays to sample tissue comes from the successful origins of XRF sensing, where the concentration of trace metals such as Fe, Zn, Cu, Hg, and Se are quantified within tissue by detection of the secondary characteristic x-rays emitted when irradiating the sample with an excitation of incident x-rays or gamma rays.^6^^–^^8^ In this case, the word “fluorescence” is used to imply the emission of these specific x-rays, having narrow energy bands specific to the inner electron shell transitions of the metals. The actual interaction between the x-ray beam and the background medium produces a dominance of Compton scattered photons, which contribute mostly to background. XRF imaging has been developed as a CT as well,^9^^–^^12^ and more recently extensively developed as a microscopy tool for sliced tissue imaging of trace elements.^13^ One of the most striking things about XRF is that while there is high specificity to metals, the sensitivity is poor when compared to most *in vivo* molecular imaging tools. The limits of detection are near 0.1  mg/g, placing this near the high millimolar range of sensitivity, while for comparison both fluorescence and nuclear medicine methods have concentration sensitivity of nanomolar to picomolar.^8^ As with optical methods, the sensitivity in thin tissues can be substantially higher than with *in vivo* use.^14^^,^^15^ The issues that limit detection in XRF are the (i) high background from Compton scatter, (ii) high detector noise, and (iii) low detector capture efficiency of the signals. Tissue sensing and imaging nearly necessitate the use of monoenergetic gamma rays or synchrotron x-ray sources to suppress the non-specific background and achieve higher signal to noise.^16^^,^^17^ The strength of the synchrotron approach is that the background is low and most of the stronger emission lines are clearly separated from one another.

From these origins in XRF over a few decades, an exciting direction of development was to utilize other excitation modes, or other emitted signals, that might provide molecular-specific signals. Additionally, these developments might provide a wider range of available signals,^18^ in the transition from strictly x-ray contrast agents to other radioluminescence excitation modes. This is illustrated in Fig. 2. For any probe agent, either molecule or nanoparticle, molecular specificity can be gained in one of two ways, either directly by probing the atom/molecule/nanoparticle itself that localizes somewhere or by using molecular target specificity, where an XRF signal that is visible is from the tag on the carrier molecule. While some metal atoms can be excellent tags, higher signal levels might be achieved by use of nanoparticles that have higher cross section per particle. But more generally, this transition brings with it the need to examine which types of molecules or molecular complexes would provide sufficient or superior signal from x-ray excitation, and also which types of emission might provide maximal emissivity from tissue for high S/N detection.

A natural transition then is to utilize a more sensitive detection mode that can reach down to the single photon level, such as either isotopic gamma emission in nuclear medicine or optical luminescence methods such as fluorescence or phosphorescence, to increase the signal level and/or suppress the background and noise levels.^18^ To preserve tissue penetration, early attempts examined x-ray luminescence imaging from a range of red to infrared emitting phosphors, as this window of wavelengths has high transmission through tissue, albeit with significant diffuse scatter. Other emission windows could be the electromagnetic regions of GHz to MHz, which have exceptionally low attenuation, although this remains to be explored fully. Alternatively, acoustic emission could be used, as is widely implemented in photoacoustics. This field is called x-ray acoustic imaging or radioacoustics.^19^^–^^23^ These modes of sensing the x-ray interaction are illustrated in Fig. 2, with the incident and detection scheme on top and the physical mode of contrast illustrated on bottom. It is interesting, albeit hard, to compare the sensitivities of detection for each of these four possible modes of detection, because the range of interactions and the range of detectors are so diverse. The interaction sensitivity for x-ray acoustic is generally low because the total amount of energy released by the radiation dose in tissue is low. However, ultrasound transducers are exquisitely well developed with high sensitivity, and so some preliminary studies have shown that this mode of detection is possible. Still the sensitivity of fluorescence emission detection can be orders of magnitude higher than acoustic detection in the setting of biological imaging, because of the ability of the detectors to capture single quanta of emission in optics. But the geometry and design of any detection system can alter this sensitivity by orders of magnitude.

**Fig. 3:**
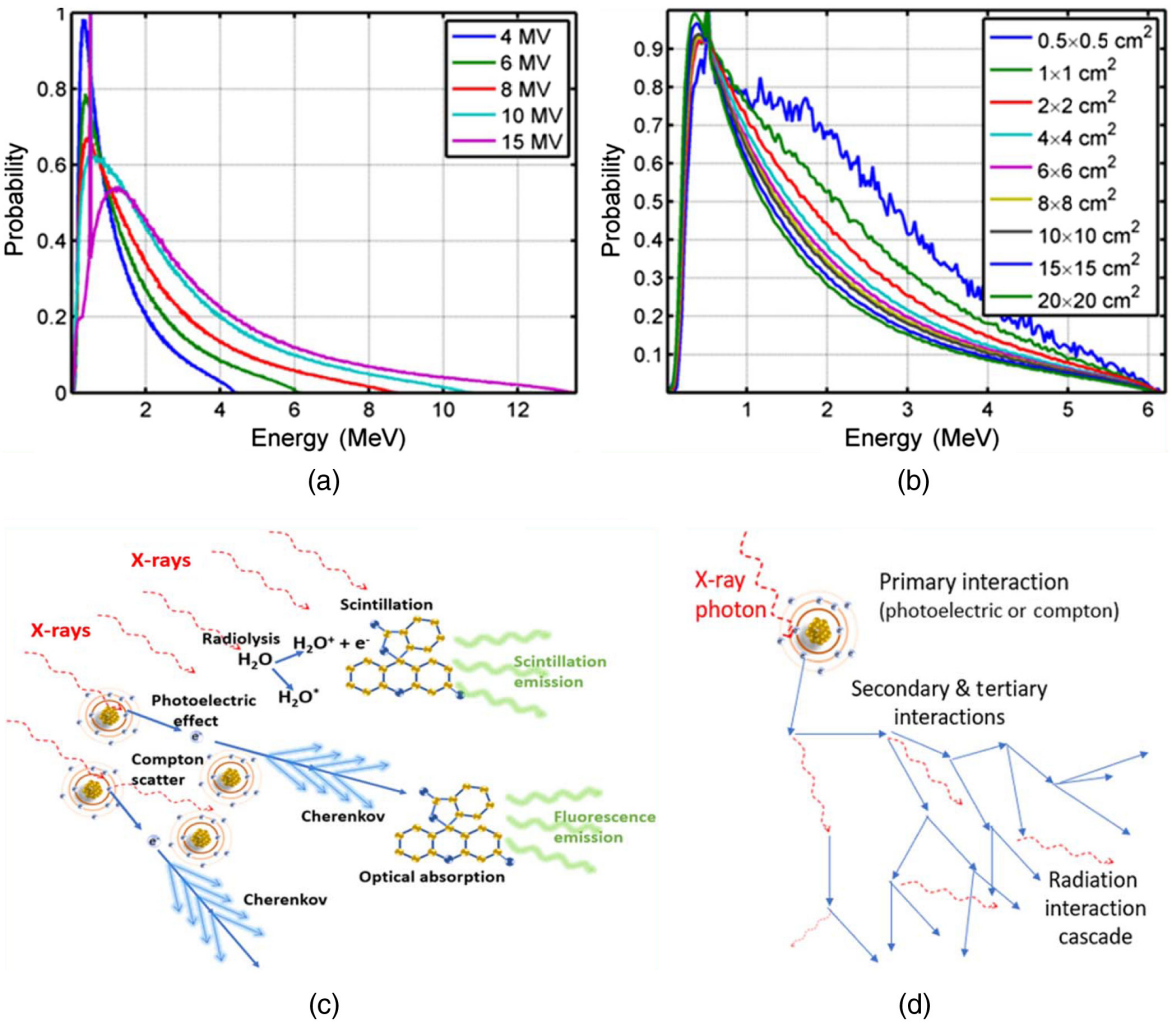
X-ray energy spectra from Bremsstrahlung production via a Linac (a) for different energies, and the spectrum for 6 MV at different beam diameters (b) showing higher energies at smaller beams. A schematic of the processes from x-rays to optical emission is shown in (c) with either direct scintillation of a molecule mediated by a variety of radiolytic events in the medium or indirect transfer by a sequence of photoelectric or Compton emitted electrons generating Cherenkov light, which then yields optical photons absorbed by the molecule. The radiation-induced cascade is illustrated in (d) from a series of interaction events between secondary electrons and photons, leading to broad mechanisms of energy dissipation in the medium.

The excitation mode of an optically active contrast agent depends upon the mechanism of interaction with the radiation, as illustrated in Fig. 2, and the energy spectrum of the radiation source. While a synchrotron or isotope sources can produce monoenergetic beams of x-rays or gamma rays, most high yield practical sources of x-rays are via the Bremsstrahlung effect from an electron beam impinging upon a target, producing a broad spectrum source, as illustrated in Figs. 3(a) and 3(b). Only a small fraction of the energy is given off as x-rays, and the spectrum is heavily weighted to the lowest energy photons. Thus, there is little specificity in the beam excitation from traditional x-ray sources, with a broad energy spectrum. The major modes of excitation, as illustrated in Fig. 3(c), are either:

1. Direct or indirect electronic excitation of a scintillator through the upper singlet states of pi electrons, resulting in eventual radiative decay. The complexity of processes depends upon the nature of both medium and scintillator, which determines their interactions. The end products of water hydrolysis (i.e., $H^{*}$, ${\mathrm{HO}}^{*}$, ${\mathrm{OH}}^{-}$, $H_{3}O^{+}$, $H_{2}$, and $H_{2}O_{2}$) dominate the interactions since water is in the largest concentration, and these can transfer energy with the scintillator. A well-known example of this is quinine, which appears to exhibit both scintillation and fluorescence; however, the interaction with the medium tends to be the dominating factor in this induction.
2. Electron soft collisions in the medium resulting in Cherenkov emission as part of the soft collision processes, which excites the molecules through direct singlet state absorption. This is proportional to the index of refraction of the medium for Cherenkov emission and then the overlap of the Cherenkov spectrum with the absorbance of the molecules. Almost all molecules with an absorption band in the visible with high emission yield would work for this method, although red absorbing molecules are more significant due to the blue absorption of Cherenkov by blood in tissue.

The overall efficiency of both these modes is generally low, although molecules with high quantum yield of emission, such as fluorescein, can make this process more favorable. The radiation cascade that occurs, Fig. 3(d), leads to a wide range of interaction mechanisms that can further decrease efficiency of any single pathway. Still the energy of a single x-ray photon (1 MV) is $10^{6}$ higher than that of an optical photon (1 eV), indicating that even a small efficiency process could lead to tens or hundreds of optical photons per x-ray photon.

## 3 X-Ray-Induced Optical Tomography

X-ray luminescence tomography was first postulated in 2010, showing that radioluminescent particles could be imaged from within tissue phantoms by scanning x-ray beams to excite them and capturing the emission of the signal.^4^^,^^24^ This was advanced to include modeling of the diffusive light transport, accounting for signal loss as a function of distance between the emission site and the light detection at the surface. The use of a partial angle approach to imaging is almost required for this type of work and several groups illustrated how this could be achieved in phantoms^25^^–^^29^ and *in vivo*.^30^^,^^31^ The major limitation in this aspect of work has been the sensitivity to mainly nanoscintillators used. The interaction mechanisms explored have largely been via direct excitation of the particle by the x-ray beam, where the reliance upon the photoelectric effect necessitates the use of high atomic number, Z, materials, and largely crystalline structures to gain sufficient radioluminescence yield from the process (see Fig. 2). The efficiency depends significantly upon the energy of x-rays, E, used because of the strong interaction cross section dependence of the photoelectric effect with energy, being $\sigma \approx Z^{4}/E^{3}$.

In comparison to direct x-ray interaction, the process of Cherenkov radioluminescence provides a secondary mechanism for excitation. The strength of using Cherenkov is that it is produced throughout the volume, proportional to dose, and provides a broadband blue-white light source within the tissue. Although the weaknesses are that it is only produced from secondary electrons above the threshold of 220 keV energy in tissue and with a yield about 1% of the total dose delivered. So, while there are attractive optical features of this excitation, it requires high-energy x-ray sources and yet produces a limited yield of light. Still, Cherenkov luminescence imaging has been demonstrated from isotopes *in vivo* as well as from linear accelerators used in radiation therapy. The latter provides a way to image tissue with higher fidelity because of the ability to scan the beam across the tissue and use image processing tools to recover high-resolution images. The major benefit of this approach is that fluorescent and phosphorescent excitation can be achieved directly by absorption of the Cherenkov light by the molecular probe. These molecular probes are smaller, and many can be biocompatible, as are discussed as follows.

## 4 X-Ray Sources, Beams, and System Design

### 4.1 X-Ray Sources and Beam Control

One of the key values in x-ray-based molecular sensing is the concept that the position of the excitation source is known *a priori* and can be used in an image reconstruction process, such that even if the emission is blurred by optical scattering through tissue, there can be a high fidelity image recovered. This was initially demonstrated in x-ray optical luminescence tomography by Pratx et al.,^3^ using keV x-ray imaging and image reconstruction. Diffusion modeling can be employed to reduce errors associated with the intensity of the luminescence emission being reduced by the tissue propagation. The geometries used directly follow those available for x-ray CT because the source technology is derived from this. In the keV energy range, swept line scan beams or partial angle tomography is possible,^12^^,^^29^^,^^32^ and in MV photon beams from linear accelerators, multi-leaf collimators (MLC) and jaws are available to shape the beams dynamically.^33^ Full field imaging can work as well, albeit without significant axial resolution because of optical scatter in the tissue, but providing apparent high resolution in lateral imaging of the tissue surface.^34^

### 4.2 Temporal Acquisition

The first studies of x-ray-induced optical luminescence based upon scintillation did not acquire in temporal sampling because the x-ray sources were continuous, and so there was no inherent value to temporal sampling. However, in Cherenkov-excited luminescence imaging, there is both inherent value in removal of the background Cherenkov excitation light, as well as the benefit of lock-in detection to the pulsed x-ray source. Linear accelerators produced for clinical use most commonly have pulsed electron bunches that are accelerated in short 3- to 5-ms pulses, with a low repetition rate near 100 to 400 Hz. This sampling is significantly slower than used for time-resolved fluorescence but fits with time-resolved phosphorescence methods, and so the first *in vivo* demonstrations have focused on imaging luminescence from oxygen sensors that have triplet states that are quenched on this timescale. Faster pulsed x-ray sources exist though, such as small x-ray sources for portable x-rays^20^ or even large sources used for industrial pulsed radiography.^35^ The fastest commercially available pulsed x-ray sources are in the tens of nanoseconds range for pulse length, and the completeness of the fall off is usually uncharacterized or unspecified. Thus, fast time gating for things such as fluorescence from organic molecules, with lifetimes usually in the nanosecond range, would need a deconvolution for the x-ray-detector instrument response function,^36^^,^^37^ as is commonly done in time-correlated single photon counting work.^38^

Temporal gating has been demonstrated with luminescence emission to suppress the Cherenkov light signal and allow for a nearly background-free sensing of the tissue. Single lymph node imaging was demonstrated by Zhang et al,^33^ using an oxygen sensing agent, and the lifetime recovery between 22 and 44 ms provided the ability to sense the local partial pressure of oxygen (${\mathrm{pO}}_{2}$). Sensing of other luminescent species is readily possible and Europium microspheres are commercially available agents for binding with targeting moieties that have a luminescence lifetime in the 100’s of microseconds.^39^ Alternatively, silicone nanoparticles also have a long lived lifetime and can be used as a light signal generator with targeted delivery.^40^

### 4.3 Lateral and Axial Spatial Resolution

**Fig. 4:**
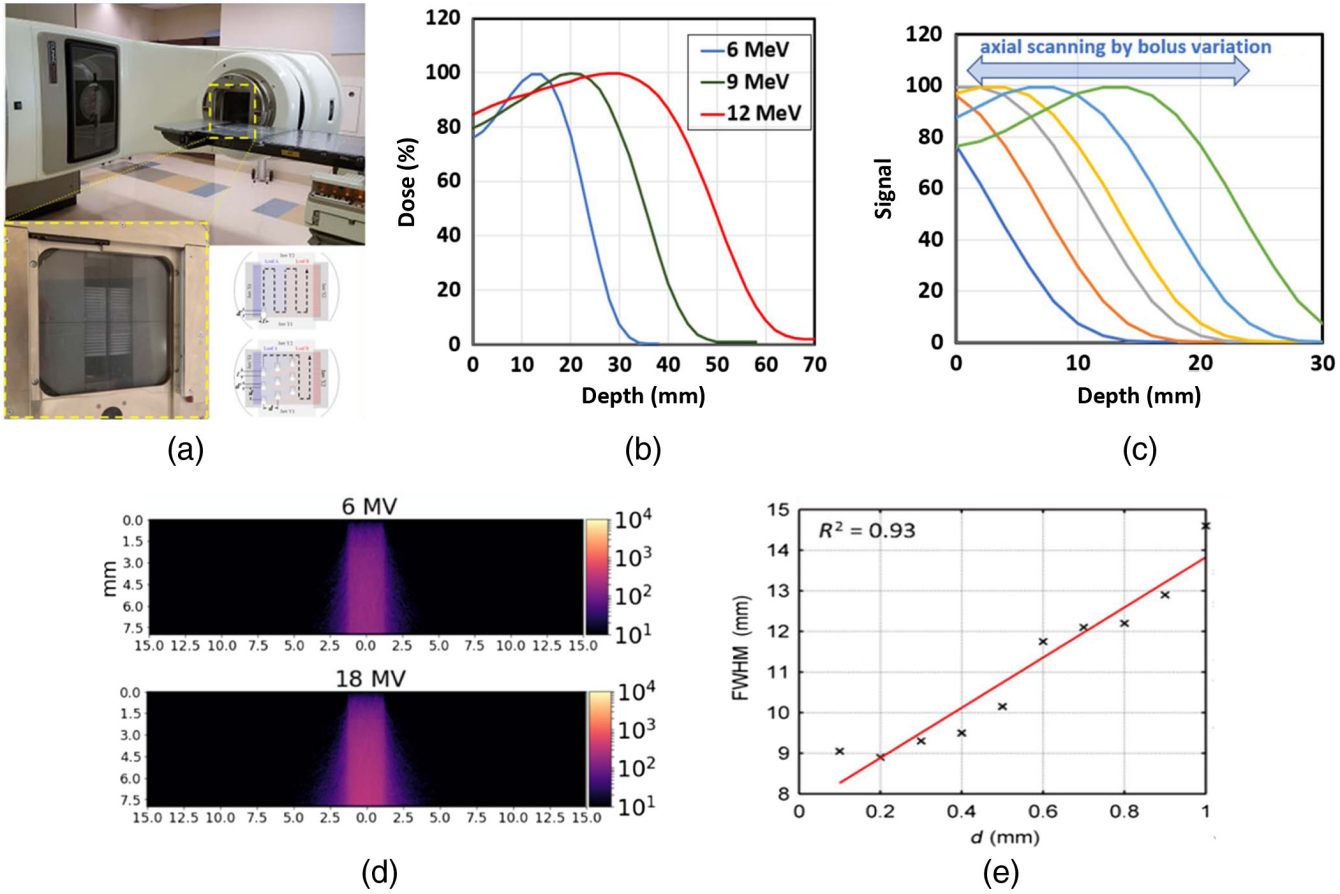
The lateral spatial confines of the beam in tissue are determined by the Linac (a) beam shaping by the MLCs (photo inset below) with illustration of how the MLCs can be used for point raster scanning or multipoint scanning.^41^ (b) The depth of sensing is affected by the choice of radiation and beam energy, as illustrated by depth–dose curves for electron beams. The scan could be axially modulated in depth by variation of buildup bolus between the tissue and the beam, as illustrated in (c). Lateral resolution is limited by the ability to slice or focus the x-ray beam, as illustrated by Monte Carlo simulations of at 6- and 18-MV beams (d), where the beam is directed downward and the X-Y axes show the mm dimensions into the tissue and the color bar is a Cherenkov intensity scale.^42^ The final lateral spatial resolution can be deconvolved with the beam width, show sensitivity to sub-millimeter objects, as illustrated in (e) where thin luminescent capillaries of varying diameter, d, were used to show sensitivity to resolve the full width at half maximum (FWHM) down to 0.2-mm diameter.^33^ The FWHM is a convolution of the beam width with d.

Perhaps one of the most undeveloped areas in x-ray-based molecular sensing is control of the x-ray source for improvement in spatial resolution. Advances in conformal and adaptive radiotherapy have led to improved tools on a Linac to control beam dose deposition. The MLCs present on the output of the Linac have advanced to a very high degree of precision, where millimeter level accuracy in dose fall off can be achieved. This same precision can then be deployed where the lateral and axial extent of the beams are used to adjust the sampling of tissue. As mentioned above, lateral resolution is largely controlled by the MLCs and jaws of the Linac, but the axial resolution is determined by choices of the beam radiation type (electrons, photons, and protons) or beam depth of scanning, as illustrated in Fig. 4(a). Thus, the Linac MLCs provide a simple technological way to shape the beams for line scans, point scans, multi-point or multi-line scans [see Fig. 4(a)], or even more generally a set of orthogonal basis functions in the source patterns for approaches such as compressed sensing.^41^^,^^43^^,^^44^

If the depth of penetration is to be restricted, it is feasible to use electron irradiation which has limited depth of penetration, and even apply a variable bolus or buildup region to the beam before the tissue, illustrated in Fig. 4(b), thereby allowing the depth of sampling to be varied externally to the Linac, just like an MLC adjusts the lateral component of the beam. The depth of sampling with electrons could be varied as they have the steepest fall off curve as compared to photons. Alternatively, in keV systems, various attempts have been shown to use x-ray focusing mirrors or fiber optic tapers^45^^–^^47^ and as this technology evolves it might be feasible to limit lateral resolution with this focusing approach.

### 4.4 Multiplexing of Signals

Excitation with x-rays implicitly leads to a broadband signal that can excite more than one molecular probe. Both scintillation and Cherenkov-excited signals can occur, and it is possible to detect multiple emission signals, based either on temporal gating or wavelength separation of intensity. The excitation of multiple molecular dyes by Cherenkov has been shown, with optimal spectral windows being in the red,^48^^,^^49^ near-infrared, and short-wave infrared (SWIR) (also referred to as near-infrared window II, NIR-II)^50^^–^^52^ wavelength bands. In particular, SWIR emitting agents are often nanoparticulate and thereby optimally suited for direct x-ray excitation.^50^^,^^53^^,^^54^ There is a high volume of research in this area, and multicolor emitters are available.^55^^,^^56^ Detectors for SWIR wavelengths are often different than those for the red or NIR wavelengths,^57^ and so it is feasible to use separated detectors for parallelization or spectral decomposition through a spectrometer.^58^^,^^59^ Time-gated detection can be coupled with wavelength-based separation of the signals as well, to further maximize the ability to detect signals at the same time, and scintillation-based signals can be orders of magnitude faster than organic fluorescence, which in turn is orders of magnitude faster than phosphorescent signals. Careful sampling of the time-sequence of the x-ray pulses with the detector gating can optimize this.

## 5 Image Reconstruction

### 5.1 X-Ray Beam Location Used as Prior Spatial Information in Imaging

X-ray photons experience much less scattering than optical radiation in tissue, and therefore x-ray-induced radioluminescence largely originates within or very near the volume that is directly in the pathway of the scanning beam. The limits on resolution though can be defined by the x-ray Compton scattering, which can happen both inside and outside the tissue. For keV sources, the scattering is usually not significant because it largely produces soft x-rays that have very short penetration depth in tissue. This is true both outside the tissue and within the tissue. In the MeV approach, the Linac MLC (Fig. 4) provides a simple technological way to shape the beams for various scans as mentioned above, but also to induce x-ray scattering near the leaf boundaries, to some degree, depending on the specific leaf end structure.^60^^,^^61^ A similar situation exists within the tissue, where in Cherenkov-excited luminescence emission, most Cherenkov photons (in the UV-blue spectral range) have very short diffusion path (<1  mm) in the tissue due to the hemoglobin and water absorption. These properties constitute the key aspect of the x-ray-induced molecular imaging scheme, where the distribution of optical signal along the direction of scanning can be recovered by measuring total luminescence signal and considering that the signal all originated from the position of the scan beam in the tissue, used as prior information in a reconstruction algorithm.

### 5.2 Diffusion Modeling of Cherenkov and Optical Photons

The radiative transfer of photon diffusion through turbid media can be solved either numerically through Monte Carlo simulation or approximated analytically by the diffusion approximation. Monte Carlo solutions generally maintain high accuracy and wide applicability,^62^^,^^63^ and their use has been tremendously accelerated by computation on GPUs.^64^^–^^69^ There are extensions of this to model high-energy particle transport that can also be used, e.g., GEANT4, although with considerably more computational effort.^70^^–^^73^ The diffusion approximation provides a first-order approximation of transport over distances beyond a few millimeters, and it has been broadly applied in diffuse optical tomography. The limits to this approximation though are important, meaning that it must be applied only where the detected photons are scattered sufficiently to lose their original directions. This condition could be marginally met in Cherenkov imaging depending in the red and NIR wavelength bands.^74^^,^^75^ For example, the emission UV-blue spectral range that always predominates the measurement is significantly absorbed in most biological tissues, and so is rarely diffuse in transport.^76^^,^^77^

Cherenkov light is generally modeled in a comprehensive manner that involves distinct behavior of a broadband wavelength spectrum of photons peaked in the ultraviolet and decaying in intensity with an inverse square dependence upon the wavelength.^78^ Modeling of x-ray-induced Cherenkov as the excited light contains two coupled processes of excitation and emission, and explicitly would require modeling the x-ray interactions with Monte Carlo tools such as GEANT4. In practice, the Cherenkov/x-ray beam can be assumed to be non-diffuse source of light inside the tissue, to simply the process. The Monte Carlo codes that model radiation-induced light transport in biological media have been integrated into the GAMOS interface to GEANT4.^73^

### 5.3 Typical Image Reconstructions: Advantages and Limitations

Currently, image reconstructions could be coarsely classified into three categories: model-based^79^^,^^80^ direct deconvolution,^43^^,^^81^^,^^82^ and back-projection.^32^^,^^83^ The model-based approaches have the widest applicability to measurement geometry and excitation mechanisms. However, a model-based reconstruction can be inefficient and even ineffective when the target is close to or far from the boundary at the detection side. In the former case, a stringent forward model using either Monte Carlo or higher-accuracy approximations to exact radiation transport modeling is requisite; while in the latter case, any tiny perturbation and inconsistency of the optical properties could lead to erroneous reconstructions on both the intensity and location. The recontruction can be improved by combining useful prior information about the measurment geometry, charateristic emissions, etc., into the regularization process, which has been an effective way, albeit with the assumption of the priors being accurate.^84^^–^^90^

**Fig. 5:**
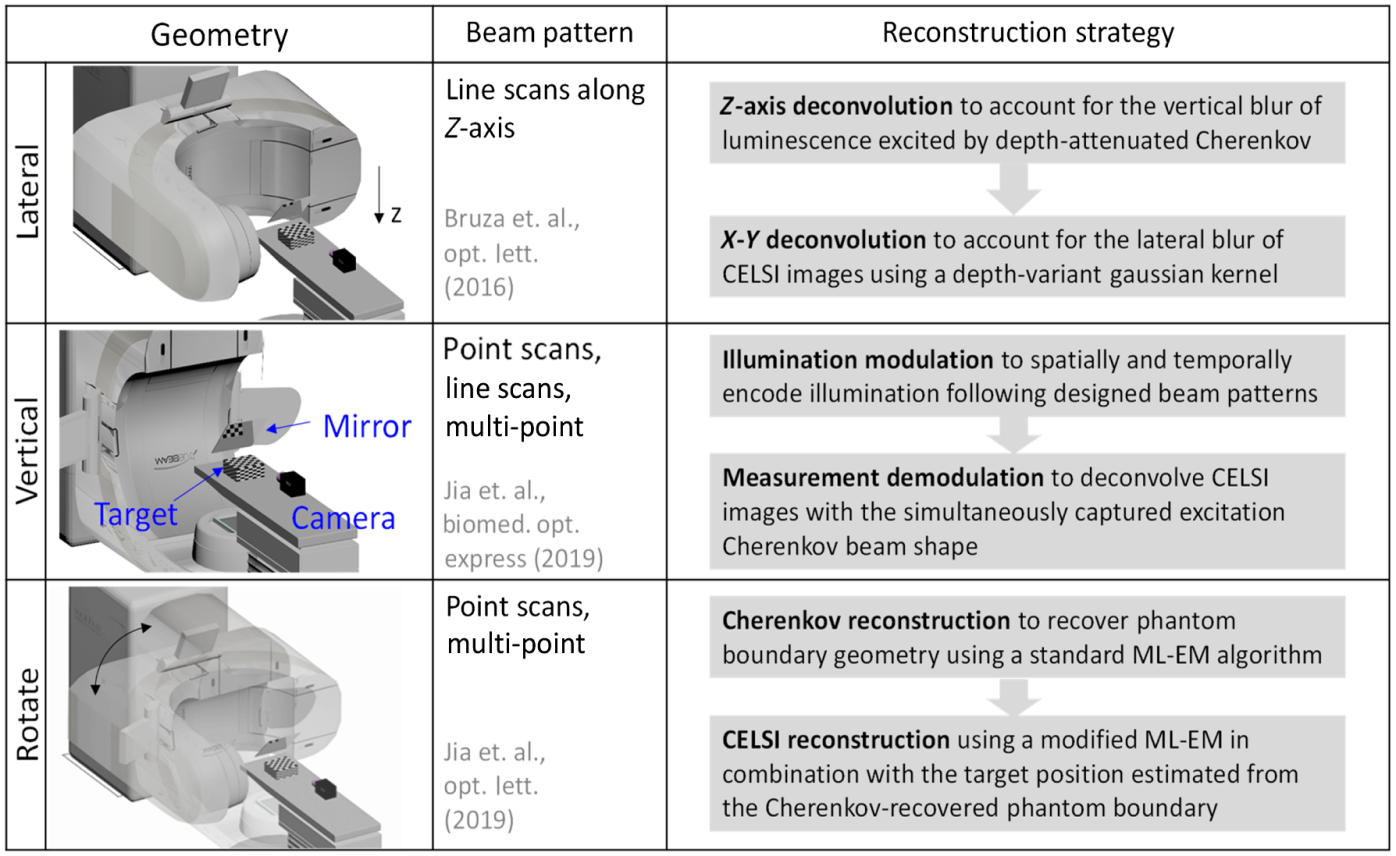
Cherenkov radioluminescence measurement geometries based on Linac MLC delivery and corresponding non-model-based image reconstructions. Laterally scanned imaging (top) sends sheet-shaped x-ray beams to measure local luminescence emission that is then deconvolved with the kernels derived from a diffusion approximation. Vertically scanned imaging (middle) spatially and temporally modulates the Cherenkov illumination following specific beam patterns and then demodulation with the simultaneously acquired excitation Cherenkov beam shapes is possible. Like the imaging technique used in CT (bottom), CELSI images were acquired by rotating the Linac gantry to yield a sinogram, which was then used with FBP of the data for image reconstruction. In all experiments, a plane mirror was always used to avoid direct exposure of the imaging camera to the radiation beam.

By taking advantage of a careful measurement geometry and utilization of coded illumination techniques, a straightforward direct deconvolution has been used for reconstruction, e.g., Cherenkov-excited luminescence scanned imaging (CELSI). CELSI uses the collimating system [Fig. 4(a)] of a Linac to send a sheet of radiation traveling across the imaged subject in a manner equivalent to the excitation-beam shaping used in light-sheet microscopy, as illustrated in the first row of Fig. 5. By restricting the excitation beam to a single, narrow sheet, the origin of the optical photons can be inferred regardless of where these photons were detected or how many times they scattered in tissue. Direct deconvolution was also applied for a vertical illumination geometry as shown in the second row of Fig. 5. By simultaneously capturing both Cherenkov and CELSI images that were excited via spatially modulated x-ray beam, the image recovery was improved by a spatial demodulation strategy at each time step based upon compressed sensing techniques. However, direct deconvolution typically suffers from two issues: (i) accurate convolutional kernels are generally hard to be determined with acceptable computational cost and (ii) numerical deconvolution could magnify the measurement noise when the amplitude approaches zero in the frequency domain.

Backprojection or filtered backprojection (FBP) reconstruction algorithm originally developed for CT has been used, known as x-ray luminescence CT (XLCT).^3^^,^^91^ Like the measurement geometry in CT, raw images in XLCT were acquired by rotating the Linac gantry (see the last row of Fig. 5) to render a sinogram that was used in backprojection-based image reconstruction. Each element in the sinogram is an integration through the pixels of a captured image, essentially implementing single-pixel imaging that facilitates the use of single-photon point detector for high-sensitivity measurement in conjunction with the TCSPC technique and the use of a spectrometer for hyperspectral studies. In XLCT, anatomical prior information can be provided by either x-ray imaging or Cherenkov surface data.^44^^,^^92^^–^^94^ Since the measurements are taken from multiple views, XLCT *in-vivo* imaging is similar to CT imaging in that the image is always better with more radiation, which can lead to excessive radiation dose, and so care must be taken to design the scan sequence. To address this issue, a lot of effort has focused on sparse-view XLCT by taking advantage of improved measurement geometry and algorithms.^92^^,^^95^^,^^96^

## 6 Radioluminescent Reporters: Scintillators and Fluorophores

The range of x-ray choices, as described already, also affects the radioluminescent reporter agent that is optimally used, as the sources in the keV range excite scintillators exclusively, whereas the sources in the MeV range can excite all of fluorophores, phosphors, and scintillators.

Development of scintillating nanoparticles that can convert x-ray radiation into UV–vis–NIR light is a very active area of research. While there is incredible promise, all known scintillating reporters are in preclinical molecular imaging research or *ex vivo* use.^97^ With slight scattering and absorption of x-ray in soft tissues, this type x-ray-excited luminescence allows for deep *in vivo* optical imaging with ultrahigh spatial resolution and negligible tissue autofluorescence.^4^^,^^98^ Lanthanide-doped fluoride-based nanoparticles have a high atomic number and proper electronic energy states for downconversion of x-rays into UV–vis–NIR luminescence.^98^^–^^101^ $\mathrm{NaGdF}4:{\mathrm{Eu}}^{3+}/{\mathrm{Tb}}^{3+}$ nanoparticles are a representative type, with efficient luminescence emission under x-ray irradiation, because the emission energy transitions within ${\mathrm{Gd}}^{3+}$ can resonantly couple to the excited state of ${\mathrm{Eu}}^{3+}/{\mathrm{Tb}}^{3+}$ ions in the ${\mathrm{Gd}}^{3+}\text{-}{\mathrm{Eu}}^{3+}/{\mathrm{Tb}}^{3+}$ host–dopant combination.^102^^–^^104^ Lanthanide-doped oxide-based nanoparticles also show bright x-ray-excited luminescence.^105^^–^^108^ For example, ${\mathrm{Gd}}_{2}O_{2}S:\mathrm{Tb}$-based nanoparticles have been designed for pH-dependent sensors, for monitoring bacterial infection or as nanocapsules that carry chemotherapy.^109^^–^^111^ Some scintillating nanomaterials in this category exhibit x-ray-excited persistent luminescence, which can still emit long-lasting phosphorescence after the x-ray irradiation is completed.^106^^,^^112^^,^^113^ Other composite nanomaterials, such as quantum dots (QDs),^114^ silicon nanocrystals,^115^ metal–organic structures,^116^ and gold nanoclusters,^117^ have also been reported to emit luminescence under x-ray irradiation. A table containing a list of most molecules and nanoparticles used *in vitro* or *in vivo* is presented in Table 1 with a summary of each key discovery.

**Table 1:** *In vitro* or *in vivo* studies using or developing optical molecular probes for x-ray excitation.

	Probes	Source	Appl.	Main results	Refs.
1	Oxyphor, G4, 2P	MeV	*In vivo*	Tomographic imaging of ${\mathrm{pO}}_{2}$ in deep tissue using Cherenkov excitation with radiotherapy.	118, 119
				Partial pressure of oxygen (${\mathrm{pO}}_{2}$) in a rat lymph node was imaged by Cherenkov-excited luminescence scans.	120
				*In vivo* oxygenation imaging, defining the resolution, depth, and sensitivity limits for Cherenkov excitation scans.	80
				*In vivo* mapping of tumor ${\mathrm{pO}}_{2}$ distributions with sub-mm spatial resolution and tracking response to radiotherapy.	34
2	Eu chelate microspheres	MeV	*In vitro*	High-resolution Cherenkov-excited luminescence scanning imaging during a standard dynamic radiotherapy.	43
			*In vivo*	Tomographic Cherenkov-excited luminescence via multi-pinhole scan approach for high-resolution *in vivo* imaging.	43
3	IRDyes 680RD, 700DX and 800CW	MeV	*In vivo*	Cherenkov-excited fluorescence of NIR IRDyes was successfully detected by spectrally resolving approach.	58
4	PdSe QD	MeV	*In vivo*	Cherenkov-excited SWIR, 1000- to 1700-nm fluorescence imaging using long Stokes-shift PdSe QDs.	52
5	${\mathrm{LaF}}_{3}:{\mathrm{Ce}}^{3+}$, ${\mathrm{Tb}}^{3+}$ and ${\mathrm{LaF}}_{3}:{\mathrm{Tb}}^{3+}$	keV	*In vitro*	Luminescence dominated by emission from ${\mathrm{Tb}}^{3+}$ ions and enhanced by organic surface coating.	99
6	Aerogel and Sylgard 184	keV	*In vivo*	Luminescence of composite silica aerogels and Sylgard 184 and ${\mathrm{La}}_{2}O_{2}S:\mathrm{Eu}$ phosphor in subcutaneous detection.	105
7	${\mathrm{Sr}}_{2}{\mathrm{MgSi}}_{2}O_{7}:{\mathrm{Eu}}^{2+}$, ${\mathrm{Dy}}^{3+}$	keV	*In vitro*	Persistent luminescence where characteristics are highly associated with the synthesis conditions.	121
8	${\mathrm{HfO}}_{2}:\mathrm{Eu}$ nanoparticles	keV	*In vivo*	Bioinert nanoparticles for biological luminescence imaging with excitation by x-rays and UV-visible radiation.	122
9	${\mathrm{NaGdF}}_{4}:\mathrm{Tb}@{\mathrm{NaYF}}_{4}$	keV	*In vivo*	Immunoassay tags for autofluorescence-free high-sensitivity detection of alpha-fetoprotein biomarkers.	104
10	$\mathrm{ZnGa}2O4:{\mathrm{Cr}}^{3+}$	keV	*In vivo*	Delayed emission up to 6 h, at 700 nm for *in vivo* whole body and tumor imaging.	106, 112
11	${\mathrm{NaLnF}}_{4}:\mathrm{Tb}@{\mathrm{NaYF}}_{4}$ with BHQ1-DNA	keV	*In vitro*	Nanocrystal scintillator-based aptasensor to selectively sense lysozymes in serum samples through FRET.	101
12	${\mathrm{Lu}}_{2}{\mathrm{SiO}}_{5}:\mathrm{Ce}$ with AlNap and AlRhod	keV	*In vitro*	Luminescence tuned from blue to green and red using FRET and able to be successfully imaged *in vitro* with rat cortical neurons.	107
13	$\beta \text{-}{\mathrm{NaGdF}}_{4}:X\%$${\mathrm{Eu}}^{3+}$	keV	*In vivo*	Nanoparticles modified for high luminescence intensity and ultralow cytotoxicity, for *in vivo* x-ray luminescence CT.	91
14	${\mathrm{NaLuF}}_{4}:\mathrm{Gd}$, Eu@Gd, Lu@Gd, Lu, Tb	keV	*In vitro*	Excited by x-ray radiation for deep tissue PDT and optical imaging, low dark toxicity and effective photocytotoxicity.	100
15	${\mathrm{Gd}}_{2}O_{2}S:\mathrm{Eu}$ scint-based pH sensor	keV	*In vitro*	An x-ray-excited luminescence-based pH sensor was fabricated to monitor bacterial infection and treatment of implanted devices.	110, 111
16	Au25-BSA cluster	keV	*In vitro*	The x-ray-excited optical luminescence of biomolecule-directed metal clusters demonstrated.	117
17	Hf-MOF and Zr-MOF	keV	*In vitro*	Two metal organic frameworks (MOFs) to efficiently convert x-ray to visible-light luminescence were designed.	116
18	$\mathrm{PEG}\text{-}{\mathrm{SrAl}}_{2}O_{4}:{\mathrm{Eu}}^{2+}$	keV	*In vivo*	*In vivo* optical bioimaging in deep tissues using soft x-ray-activated persistent luminescence.	113
19	${\mathrm{NaGdF}}_{4}:{\mathrm{Eu}}^{3+}$	keV	*In vivo*	X-ray-excited luminescence and photoluminescence, characterization of crystal structure and extrinsic factors.	103
20	${\mathrm{Sr}}_{8}({\mathrm{Si}}_{4}O_{12}){\mathrm{Cl}}_{8}:{\mathrm{Eu}}^{2+}$	keV	*In vitro*	Temperature-dependent radioluminescence, tested via UV light and x-ray excitation.	123
21	PEG-$\mathrm{NaGd}{\left({\mathrm{WO}}_{4}\right)}_{2}:\mathrm{Eu}$	keV	*In vivo*	Used as highly effective radio luminescent nanoprobe for x-ray optical imaging and contrast agent for MRI and CT.	124
22	Oxide-embedded Si-NCs	keV	*In vitro*	The formation and x-ray luminescent characterization of oxide-embedded silicon nanocrystals (Si-NCs).	115
23	$\mathrm{DOX}@\mathrm{Gd}2O2S:T_{b}@\mathrm{PSS}/\mathrm{PAH}$	keV	*In vivo*	Nanocapsules synthesized for measuring pH-triggered release of doxorubicin with x-rays.	109
24	${\mathrm{BaGd}}_{x}Y_{1-}{\mathrm{ZnO}}_{5}:{\mathrm{Yb}}^{3+}$	keV	*In vivo*	Highly efficient x-ray-excited SWIR luminescence phosphor for the deep-tissue biological imaging.	53
25	CdTe QDs	keV	*In vivo*	Contrast in phantom and mouse tests, quantified using clinical x-ray system at 20 and 120 keV.	114
26	${\mathrm{Cs}}_{2}{\mathrm{NaY}}_{0.99}F_{6}:0.01{\mathrm{Pr}}^{3+}$	keV	*In vitro*	Strong ultraviolet C (200 to 280 nm) emission and afterglow for >2-h post-irradiation.	125
27	${\mathrm{CeO}}_{2}:{\mathrm{Eu}}^{3+}$	keV	*In vitro*	Red light emission excited with UV light and x-ray.	108
28	${\mathrm{Ba}}_{0.55}Y_{0.3}F_{2}:{\mathrm{Eu}}^{3+}$	keV	*In vivo*	Water-soluble cubic nanophosphors surface modified for *in vivo* imaging with β+ from F18 and x-ray.	98

MeV x-ray-induced Cherenkov emission can be used as a controllable indirect light source that can scan the imaging objects to excite optical molecular probes, without the need for scintillation.^41^^,^^80^^,^^81^^,^^120^ While the overall yield of Cherenkov is low compared to scintillation, the attractive optical feature of Cherenkov is its broadband spectrum, ranging from UV through visible to NIR wavelengths,^51^ which provides potential to excite almost all the optical molecular probes with absorption in this spectral region in theory. The challenge of this though is that the broadband signal introduces a spectral overlapping problem in the optical signal detection. To unmix the Cherenkov emission and the secondary optical emission signal, phosphorescent probes with long lifetimes have been used for Cherenkov-excited luminescence imaging with time gated acquisition.^126^ Alternatively, spectrally resolved detection based on spectrometer detection can capture the Cherenkov-excited fluorescence.^58^ In addition to this, SWIR (1000 to 1700 nm) fluorophore PdSe QDs can also be used with Cherenkov-excited fluorescence imaging, to try to minimize the emission overlap based upon the longer Stokes-shift of the SWIR emitter.^52^ Perhaps the single most important feature of Cherenkov absorption is the fact that organic dyes can be used and therefore have a reasonably good potential for human use. In particular, emission is likely available from all FDA-approved fluorophores, such as fluorescein, methylene blue, and indocyanine green, although the emission quantum yield of the probe plays a big role in the efficiency of detection.

## 7 Applications and Future Directions

### 7.1 Molecular Sensing of Oxygen and pH

**Fig. 6:**
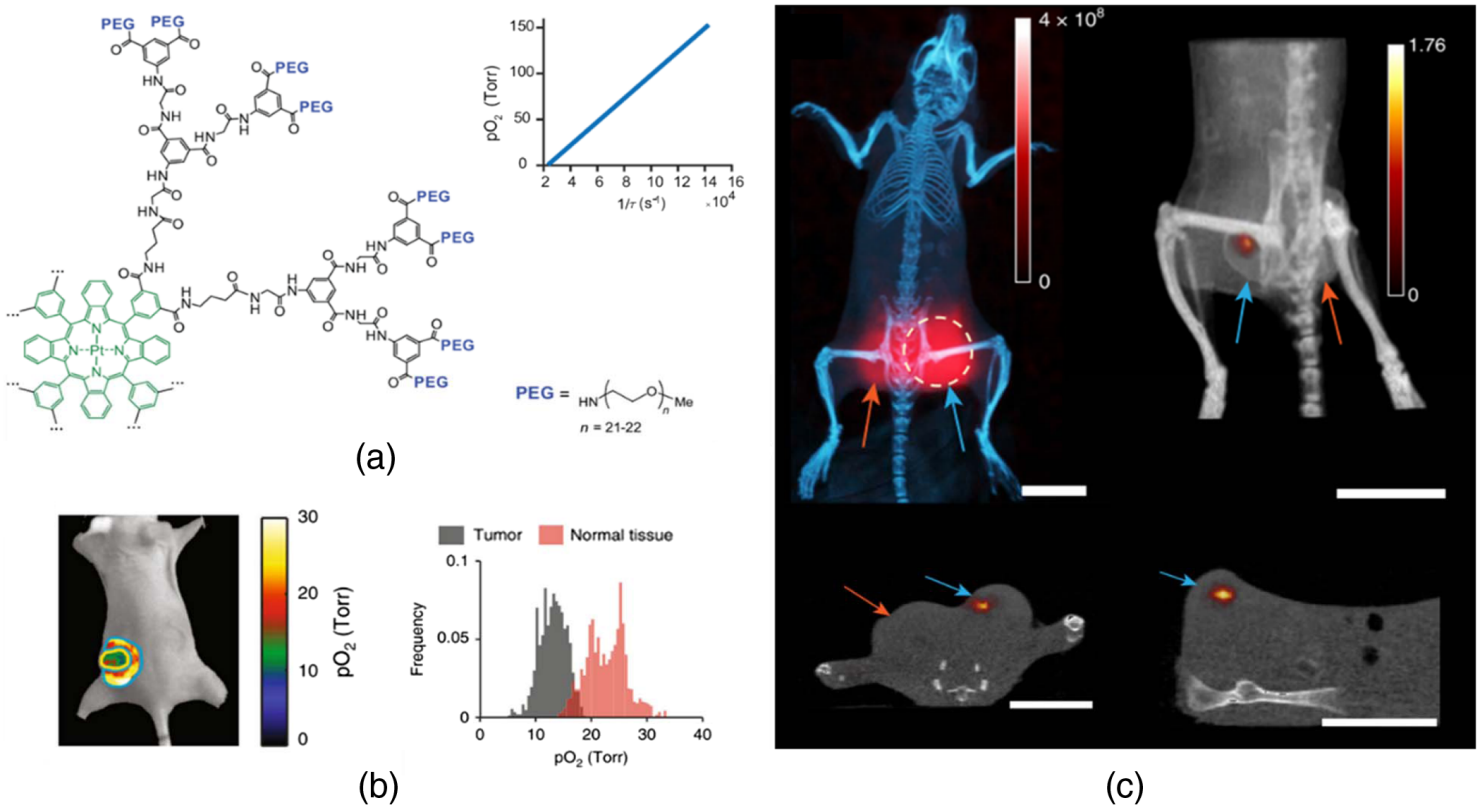
Cherenkov-excited luminescence imaging of tumor oxygen. (a) Structure and oxygen quenching property of probe PtG4.^34^ (b) *In vivo* ${\mathrm{pO}}_{2}$ imaging of tumor and normal tissue of a mouse after IV injection of PtG4.^34^ (c) Maximum-intensity projection luminescence image overlaid to x-ray CT scan and tomographic luminescence of PtG4.^80^

Tumor oxygenation significantly affects the outcome of radiotherapy, and hypoxic tissues such as tumors (defined as having partial pressure of oxygen, ${\mathrm{pO}}_{2}$, less than 10 mmHg) are known to be more resistant to radiation damage than fully oxygenated tissue.^127^^–^^129^ Therefore, monitoring of tumor oxygenation is thought to be highly desirable for effective radiotherapy. Cherenkov-based imaging provides an internal light source to excite oxygen-sensitive phosphorescence probes during radiotherapy (Fig. 6).^34^ Tomographic oxygen images were reconstructed in experimental mice, to sense the ${\mathrm{pO}}_{2}$.^118^^,^^119^ Spatial resolution was improved by spatially encoding of the beam positioning with the Linac MLCs and subsequent deconvolution of the beam width from the signal.^120^^,^^130^ Using a scanning sheet-shaped MV x-ray beam, the excitation was restricted to narrow volumes, and tomographic oxygenation imaging with sub-millimeter resolution and nanomolar sensitivity at depths of several millimeters was demonstrated (Fig. 6).^80^ An alternative approach used the MV x-ray-induced Cherenkov emission spectral changes from differences in attenuation by the blood oxygen saturation, ${\mathrm{SO}}_{2}$ values. Tissue blood ${\mathrm{SO}}_{2}$ alters the absorption of the emitted Cherenkov, by de-oxygenated blood absorbing more in the 600- to 750-nm wavelength band. The change in spectrum could be sampled, or more simply the change in broadband intensity could be tracked during radiation delivery.^131^^,^^132^

Another typical tumor microenvironment is the acidosis induced by high metabolic rate in poorly perfused regions of tumor, as a result extracellular pH in tumors is lower than in normal tissue and can be correlated with prognosis and response to treatment.^133^^,^^134^ An x-ray-excited pH sensor consisted of an x-ray scintillator film (${\mathrm{Gd}}_{2}O_{2}S:\mathrm{Tb}$ and ${\mathrm{Gd}}_{2}O_{2}S:\mathrm{Eu}$) coated in a methyl red-dyed paper, used for high-resolution pH detection in tissue. The pH was obtained by analyzing the optical spectrum through the paper after scanning with narrow x-ray beam.^135^ Then the noninvasive pH sensing was used to monitor bacterial infection and treatment of implanted medical devices through tissues after coating on implanted surfaces.^110^^,^^111^ Using a pH-triggered nanocapsule, spectral changes were sensitive to the release of doxorubicin, which can be used to track drug release in the acidic tumor microenvironment.^109^ Some pH-dependent long-lived emission luminescent probes appear promising for Cherenkov-based lifetime detection.^136^^–^^138^

### 7.2 Sensing of Immunology: Biological Imaging and Development of Combined Therapeutics

In the past few decades, the innovation of immunotherapy has revolutionized the approach to treatment of advanced cancer by means of immune cell therapy, stimulating the immune system to destroy malignant cells.^139^^–^^141^ Studies have used fluorescent imaging of NK cells within human prostate cancer xenografts^142^ and migration of T-cells to tumors.^143^^,^^144^ This fluorescent labeling technique for immune cells could be also used with x-rays to perform *in vivo* real-time monitoring of therapeutic effects for immune cell-based therapy. The relevance of this is because the combination of radiotherapy and immunotherapy is now shown to enhance the induced systemic anti-tumor response and achieve higher tumor control effect.^145^ Imaging has the potential to monitor immune signals during fractionated radiotherapy for patient specific sensing of the synergy between these treatments and their timing.

### 7.3 Theranostics: Molecular Sensing with X-Ray-Induced Photodynamic Therapy

The use of light as an activation mechanism has a long history in therapeutics, perhaps the most common being UV treatment for psoriasis^146^^–^^149^ and photodynamic therapy (PDT).^150^^–^^152^ PDT is a noninvasive cancer treatment modality that utilizes photosensitizers to generate cytotoxic reactive oxygen species when activated by light of appropriate wavelengths.^153^^,^^154^ PDT has its core strength in the shallow tissue penetration of illumination light, and this is widely utilized in dermatology for superficial skin lesion treatment.^155^^–^^158^ However, treatment of deep lesions has been limited by blood attenuation of the light.^159^^–^^161^ Using x-rays as the irradiation source for PDT is a way to overcome this problem. This has been extensively studied in recent years, with many approaches using nanoscintillators to convert the x-rays to UV/visible light, which activates photosensitizer deep in tissues,^162^^,^^163^ and examples with direct Cherenkov activation are possible.

This approach to x-ray-activated photodynamic therapy (XPDT) makes it feasible to integrate diagnosis and tumor therapy for tumor theranostic applications. A theranostic mesoporous silica nanoparticle encapsulated a photosensitizer, 2,3-naphthalocyanine and a ${\mathrm{LiGa}}_{5}O_{8}:\mathrm{Cr}$-based nanoscintillator was designed to efficiently mediate deep tissue XPDT and guide the irradiation by x-ray-excited luminescence imaging.^164^ This nanoparticle still produces O21 from the long and intense afterglow luminescence of ${\mathrm{LiGa}}_{5}O_{8}:\mathrm{Cr}$ after x-ray irradiation. After conjugation with Cetuximab (i.e., antibody to the EGF receptor), the nanoparticles were able to selectively accumulate in EGFR expressing orthotopic lung tumors for both EGFR-mediated molecular sensing with x-rays and XPDT. Another example constructed an x-ray-excited core–shell–shell theranostic scintillator nanoparticle based on lanthanides-doped ${\mathrm{NaLuF}}_{4}$, which could emit visible light, and was used with rose Bengal photosensitizer for PDT and imaging.^100^ Alternative approaches to drug release have used poly(lactide-co-glycolide) polymeric nanoparticles incorporating a photosensitizer (verteporfin) that could be triggered by 6-MeV x-rays to generate O21. In addition, targeting of nanoparticles with folic acid enables specific targeting of tumors that overexpress the folate receptors. Inclusion of radiation-activated ${\mathrm{TiO}}_{2}$ nanoparticles has been shown to have therapeutic effect, postulated to be mediated by Cherenkov light causing a photocatalytic effect, leading to radical species production.^165^

X-ray deposited chemotherapies have been examined in several delivery moieties. Delivery via vitamin B12 uptake via the transcobalamin receptor was shown for delivery of agents and photorelease via the alkylcobalamin scaffold that is light sensitive.^166^ X-ray activation was shown to release doxorubicin bound in micelles, by breakage an o-nitrobenzyl linker, thereby breaking open micelles, and resulting in delivery of doxorubicin to the nucleus.^167^

While this last section has focused on therapeutic effects from x-rays mediated by optical signals, there is a linkage to diagnostic scanning that could be very important, both scientifically and practically. As these therapeutic studies advance, it seems inevitable that the diagnostic potential for their use will become more apparent as well.

## 8 Conclusions

Optical molecular sensing from x-ray excitation describes a range of technologies and research studies where an incident x-ray beam is used for deep tissue sensing. The common theme is that through excitation by x-rays and active scanning or active delivery of molecular probes fundamentally new biological information could be sampled from tissue deeper than before and with higher spatial resolution. The field involves the intersection of (i) molecular probes that have high potential for radioluminescence or interaction with radioluminescence, (ii) x-ray technologies that provide specific energy, lateral or axial control, and scanning, and (iii) biomedical needs where there is not good potential for diagnostic information already. The strengths are in the widespread application and acceptance of x-rays as a diagnostic tool and the diversity of systems, energies, and controls that are well understood and developed. The challenges remain in the understanding and refinement of molecular probes that intake excitation energy from x-ray origins and maximize the output of optical signals in a way that retains meaningful molecular information by the location, intensity, or lifetime. While this field is not well defined as a single entity, it is inevitable that research will continue to define what is possible and that niche uses will become adopted, such as oxygen sensing, pH sensing, receptor uptake of nanomaterials, or X-PDT applications.

## Code, Data, and Materials Availability

This work was a review of published material and so was not completed with any code, data, nor materials.
